# Evaluating the effectiveness of a combined approach to improve utilization of adolescent sexual reproductive health services in Kenya: a quasi-experimental design study protocol

**DOI:** 10.1186/s12978-019-0825-3

**Published:** 2019-10-29

**Authors:** Lilian Mutea, Susan Ontiri, Sheila Macharia, Meital Tzobotaro, Carolyne Ajema, Vincent Odiara, Francis Kadiri, Solomon Orero, Mark Kabue, Kristien Michielsen, Peter Gichangi

**Affiliations:** 1 US Agency for International Development (USAID), Nairobi, Kenya; 2 International Centre for Reproductive Health, department of Public Health and Primary Care, faculty of Medicine and Health Sciences, Ghent University, Ghent, Belgium; 3 Jhpiego, An affiliate of John Hopkins University, Nairobi, Kenya; 4 Population Services Kenya, Nairobi, Kenya

**Keywords:** Adolescent, Sexual and reproductive health, Contraception, Family planning, Approach

## Abstract

**Background:**

Access to and utilization of adolescent sexual and reproductive health (ASRH) services remains poor. ASRH services in Kenya are primarily offered in health facilities and include counselling, information, and services on family planning, sexually transmitted infections, and HIV and basic life skills. The Ministry of Education also provides age-appropriate sexual and reproductive health information in schools. This paper presents a study protocol that will evaluate the effectiveness of a combined approach toward improving utilization of ASRH services.

**Methods:**

This will be a quasi-experimental study utilizing qualitative and quantitative methods. During the formative phase, data will be collected through focus group discussions, in-depth interviews, and key informant interviews to explore the barriers and facilitators of provision and utilization of ASRH services. A quantitative design will be used to obtain baseline and endline data through household surveys and client exit interviews. Following the formative and baseline household and client exit assessments, an intervention focusing on provision of ASRH service package targeting boys and girls will be implemented for 18 months. The package will include contextualized ASRH services, including counselling and age-appropriate, comprehensive sexual education for behavior change with an aim to increase utilization of ASRH services. An analysis of the primary outcome (utilization of ASRH services) will be undertaken to establish the difference in difference between the control and intervention arm, before the intervention (using the baseline survey data) and after the intervention (using the endline survey data).

**Discussion:**

Adolescents have now been included in the World Health Organization’s *Global strategy for women’s, children’s and adolescents’ health* (2016–2030), acknowledging the unique health challenges facing young people and their pivotal role as drivers of change in the post-2015 era. This study will generate evidence on whether a combined school, facility, and community approach works toward improving utilization of ASRH services. The information generated from the study will be beneficial for programming as it will identify underlying reasons for low utilization of ASRH services. Results will help to shape ASRH programs and reduce teenage pregnancy within Kenya and other similar low middle-income countries.

**Trial registration:**

The study is registered at http://www.pactr.org/, registration number PACTR201906738029948.

## Plain English summary

Pregnancy among girls aged 10–19 years is a problem in Kenya. While there are sexual and reproductive health services in place, adolescents’ access and use of these services remain very low. The Ministry of Health has developed four ways to promote the delivery of sexual and reproductive health services to adolescents. These include promoting adolescent access to services within community settings, health facilities, schools, and through digital platforms. We present a study protocol that will establish how this model can best reach the adolescents in two districts with a high burden of teenage pregnancies. The 18-month study will be implemented in two phases. The first phase will be to find out how aware adolescent boys and girls are of the available sexual and reproductive health services, what makes it difficult for them to use these services, and what needs to be done to improve their ability to use the services. Based on the information gathered, we will implement an intervention for 18 months that will establish the effectiveness of the model developed by the Ministry of Health in increasing adolescents’ utilization of sexual and reproductive health services. The information gathered from this study will be useful in shaping programs that aim to address adolescent sexual and reproductive health in Kenya and other developing countries.

## Background

The World Health Organization defines “adolescents” as individuals in 10–19 years old and “youth” as 15–24 years old [1]. Together, adolescents and youth are referred to as young people, encompassing the ages of 10–24 years. There is a gap in adolescents’ access to sexual and reproductive health (SRH) services and information, which has not been fully addressed. Several studies have assessed what works to increase access and utilization for SRH among young people. There is, however, a lack of scientifically sound data on the effectiveness of services that target young people in sub-Saharan Africa, especially in comparison to the magnitude of adolescent SRH (ASRH) challenges in the region [2]. Systematic reviews of available published literature suggest that current interventions targeting youth tend to have significant positive effects on improving their knowledge, and sometimes attitudes, regarding sexual behavior, but are less effective in demonstrating change in sexual behavior outcomes [3]. A review of best practices used in ASRH programs established that many ASRH interventions are implemented in an uncoordinated and piecemeal fashion, hence, they do not result in positive outcomes for adolescents [3].

Kenya has poor ASRH indicators. Adolescent pregnancy is a major problem in Kenya, with a teenage pregnancy rate of 18%, and an unmet need of family planning (FP)—as measured by the contraceptive prevalence rate among sexually active, unmarried girls aged 15–19 years—of 49% [4]. It is estimated that about 13,000 girls drop out of school annually in Kenya due to early and unintended pregnancy [5]. Adolescent pregnancy also increases the risk of maternal and newborn deaths and disability, including from complications from unsafe abortion, prolonged labor, childbirth, and the postnatal period [6].

To address the poor indicators, in 2015, Kenya launched a National Adolescent Sexual and Reproductive Health Policy, which provides guidance to government ministries and partners on how to respond to ASRH needs [7]. The policy advocates for the ministries of education and health, other line ministries, the political administration, and other stakeholders for successful ASRH programs and to ensure participation of young people. In addition, it recognizes the importance of addressing ASRH needs to achieve Kenya’s development goals. Despite this legal framework, implementation of ASRH services has been weak and uncoordinated. The absence of reinforcement of ASRH policies enables administrators and service providers to impose restrictions based on their personal beliefs that prohibit youth from gaining access to essential information and services. In addition, there is limited evidence to support the effectiveness of initiatives that simply provide “adolescent friendliness” training for health workers [2]*.* A study of young people’s perception of ASRH services in Kenya showed that young people wished to see an increase in ASRH services, especially in rural areas, including the use of mobile clinics [8]. The study also suggested the need to increase awareness of available ASRH services among young people and the community in general through outreach activities in the community, schools, and churches.

The Kenyan Ministry of Health has outlined four service delivery models for ASRH interventions: a) *community based (outreach services):* services and information are offered to adolescents within the community/non-medical settings; b) *clinic based:* services and information are offered to adolescent within/based in health facilities c) *school based*: services and information are offered to adolescents within the school setting; and d) *virtual based*: services and information are offered to adolescents within the virtual space or on digital platforms [4]. Implementation of this combined model has not been fully implemented and its effectiveness has not yet been established in Kenya. We present a protocol to evaluate the effectiveness of this model in Kisumu and Kakamega counties, which have some of the highest burden of teen pregnancy in Kenya, at 22 and 15% consecutively [9].

## Objectives

The primary objective of this outcome evaluation is to establish the effectiveness of a customized combined model in improving utilization of SRH services among adolescents aged 15–19 years.

The specific objectives are to:

1. Establish the barriers to and facilitators of ASRH use by consulting adolescents, health workers, communities, and decision makers
2. Assess the level of knowledge, attitude, practices, and healthcare seeking behavior of adolescents toward ASRH services
3. Determine the effectiveness of a customized combined ASRH intervention model in improving utilization of ASRH services

## Methods

### Study setting

The study will be conducted in Kisumu and Kakamega counties, which are neighboring counties in western Kenya. Kisumu County is 342 km from Nairobi, Kenya’s capital city while Kakamega is 360 km from Nairobi. The total population of Kakamega is estimated to be 1,975,603 and Kisumu 1,212,956 with one in four people being an adolescent in both counties. Kakamega is the second most populous county in Kenya, with a high burden of teen pregnancy at 19%; Kisumu’s teenage pregnancy rate stands at 15%, the national average is 18% [9].

### Study design

This will be an 18-month mixed method, quasi-experimental study designed to evaluate the ASRH program’s interventions on the effectiveness of the combined model to improve utilization of ASRH services. Using a qualitative design, data will be collected through focus group discussions (FGDs), in-depth interviews, and key informant interviews. The participants will be purposively selected. Further, we will use exit interviews to study satisfaction among males and females 15–19 years of age who have received ASRH services from health facilities and outreaches (quantitative design). A quasi-experimental design with intervention and control groups will be employed, using a quantitative design to evaluate the effectiveness of the intervention on utilization of ASRH services.

### Study unit inclusion criteria and selection

Kisumu and Kakamega counties were selected as project areas in part because they are among the counties with the highest burden of teen pregnancy in Kenya—at 15 and 19%, respectively [9]. The study will focus on two sub-counties within Kisumu County (Nyando and Kisumu East) and two sub-counties within Kakamega County (Matungu and Navakholo). Within the identified four sub-counties, two wards have been selected in each sub-county, one as intervention (Kobura in Kisumu and Kholera in Kakamega) and the others as control (Bunyala West in Kisumu and Kajulu in Kakamega). The selection of the intervention ward is based on the poor reproductive and maternal health indicators coupled with low utilization of FP services among adolescents [9]. The wards were selected by the study team in collaboration with Ministry of Health officials after reviewing the wards’ service statistics. The intervention-comparison wards were selected to be similar with respect to sociodemographic characteristics and low utilization of FP services.

### Study population

Participants for the qualitative phase of the study will be drawn among adolescents, community members, teachers, healthcare workers, and county health managers in the two counties in the intervention area only, as the findings will be used to shape the intervention. Study participants for the quantitative phase of the study will be adolescents aged 15–19 years. The client exit interviews will target boys and girls aged 15–19 years, who have received any ASRH service at a facility or outreach point. The household survey will target girls aged 15–19 years old who haven’t had their first birth. This is because the unit of measure will be utilization of ASRH services, specifically the use of FP services for primary prevention of pregnancy.

### Sampling

#### Qualitative study

In-depth interviews will be done among adolescents, teachers, healthcare workers, and community representatives while key informant interviews will be done with the county leadership. FGDs, comprising of sessions with only boys, only girls, and a mix of both will also be held. A total of 116 participants will be selected purposefully to participate in the study (Table 1).

**Table 1:** Formative assessment sample sizes in the ASRH intervention group, Kisumu and Kakamega counties

Participant group	Approach/Method	Description of participants	# per ward	Fre- quency	Total	Focus of the interview/FGD
Adolescents age 15–19 years	IDI (12)	3 boys and 3 girls	6	2	12	To assess adolescents’ barriers, experiences, and preferences around use of ASRH services
	FGD (6)	Girls FGD (10) Boys FGD (10) Mixed FGD (5 boys and 5 girls)	30	2	60	To assess adolescents’ barriers, experiences, and preferences around use of ASRH services
Teachers	IDI (12)	3 male and 3 female	6	2	12	To explore teachers attitudes toward providing ASRH information and services to adolescents within school; personal beliefs, policy direction toward ASRH service provision in schools, and school re-entry post pregnancy; and adaptability to meet adolescent’s health needs
Community representatives	IDI (12)	Chief, Village elder, parent, youth champion, religious leader, ward administrator, police (Officer Commanding Police Division—OCPD), community health worker	6	2	12	To explore the decision-making processes around adolescent’s sexuality and use of ASRH services, gender roles, and personal beliefs, gender-based violence against adolescents, legal issues (if any)
Health workers	IDI (10)	Representatives from public health facilities: dispensary or health center and sub-county hospital; chemist/pharmacy attendant; and private facility	5	2	10	To explore health workers experiences in providing ASRH services to adolescents
County leadership	KII (10)	County directors— health, youth, education, reproductive health coordinator; high school head teacher, primary school head teacher	5	2	10	To establish the existence of policies supporting provision of ASRH information and services in various settings and level of implementation
Transcripts	62	Total participants			116	

*IDI* in-depth interview, *FGD* focus group discussion, *KII* key informant interview

#### Client exit interviews

The survey will be powered to provide estimates of clients exit among females 15–19 years of age at the ward level, who will have received ASRH services from health facilities and outreaches. For the purposes of sample size computation, we shall assume a satisfaction level with services of 50% given the lack of a priori indicators in the two counties. The assumed 50% prevalence proportion will give the maximum sample size and any satisfaction level above or below 50% will be overpowered, implying that there will be more precision in the estimates for any other levels of satisfaction. Powering the sample to detect a 15-percentage point difference between the two arms at 85% power, a 95% confidence interval, and a design effect of 1.5, will require a base sample size of 154 in each arm. After accounting for a 20% non-response and incomplete questionnaires, the sample size at recruitment will be 185 per arm in each county. The combined sample size will be 372 respondents at baseline and endline in the two counties, thus a total of 744 (372 baseline and 372 endline). In each of the sub-county where the study will be implemented, the sample size will be allocated proportionate to the number of health facilities and outreaches available.

#### Quasi-experimental design

We will conduct a cross-sectional household survey to collect quantitative data to measure knowledge, attitudes, practices, and uptake of ASRH services. The indicator used to compute the sample size was the FP services utilization rate among adolescents aged 15–19 years, using the most current data from the study areas obtained from the Kenya health information system. The FP utilization for both groups was computed and the lowest prevalence of the two was used in computing the sample size to guarantee the maximum possible sample size. An intra-class correlation of 0.03 was assumed resulting in a design effect of 1.87. The study is powered to detect a 15-percentage point difference between the two groups in each county, 95% confidence interval, with power of 80%. Furthermore, we account for a 10% loss due to possible non-responses and incomplete data. Using the parameters described, the sample size was determined to be 264 per arm (total 528) and 231 per arm (total 462) per arm in Kisumu and Kakamega counties, respectively, at each data collection period. A sample size of 990 participants will therefore be interviewed at baseline, and a similar number at endline. Community units are made up of villages, which, for the purpose of sampling, will be “cluster units.” Since the total number of units in each cluster unit and ward is known, villages will be randomly selected; eight villages in Kobura ward and eight from Kajulu ward in Kisumu County. In Kakamega, seven villages in Kholera ward and seven villages in Bunyala West ward. The sample size will be equally distributed among the selected villages in each ward. Households will be selected through systematic random sampling at an interval of 100–200 m from a particular landmark, until the required sample size is achieved for each ward. In households with multiple adolescents, the Kish method will be used to randomly select one individual from each household [10].

### Study eligibility criteria

Adolescent boys and girls aged 15–19 years:

• Living, at the time of the survey, in the study sites

• Voluntarily provides informed consent (emancipated minors/adolescents aged 18 and 19 years)

• Voluntarily provides assent coupled with parental permission for minors

### Study implementation

The study is designed to employ two design methods: a cross-sectional formative assessment (Phase 1) and a quasi-experimental study design (Phase 2).

#### Phase 1: formative assessment

A qualitative cross-sectional study design will be utilized for the formative assessment to establish the barriers, preferences, and experiences of adolescents while seeking and using ASRH only. In addition, a pre-intervention and post-intervention client exit interviews will be conducted with both boys and girls receiving ASRH services in health facilities and outreach camps in the intervention and control arms. The aim of the client exit interview will be to establish the quality of ASRH services offered at facilities and outreach points. Information gathered in both formative assessments will be used to develop interventions that will improve access to and quality of ASRH services.

#### Phase 2: quasi-experimental design

A quasi-experimental evaluation design is proposed to evaluate the rollout of the ASRH program intervention to determine the effectiveness of a combined model to improve utilization of ASRH services. In both counties, program intervention and comparison groups will be selected, while taking measures to ensure that the comparison group matches the program intervention group in general composition, such as possession of similar sociodemographic characteristics. Using the stratified cluster sampling approach, a pre-intervention and post-intervention household survey will be conducted in the two study arms targeting adolescent girls at baseline and endline. The aim of the survey will be to establish the status of adolescents’ knowledge, attitude, and utilization of ASRH services, primarily FP. Implementation of the program intervention will be population-based, targeting adolescent boys and girls aged 15–19 years in the intervention areas. An analysis of the primary outcome (utilization of FP services) will be undertaken to establish the difference in difference between the control and intervention arm, before the intervention (using the baseline survey data) and after the intervention (using the endline survey data).

### Description of intervention

**Fig. 1:**
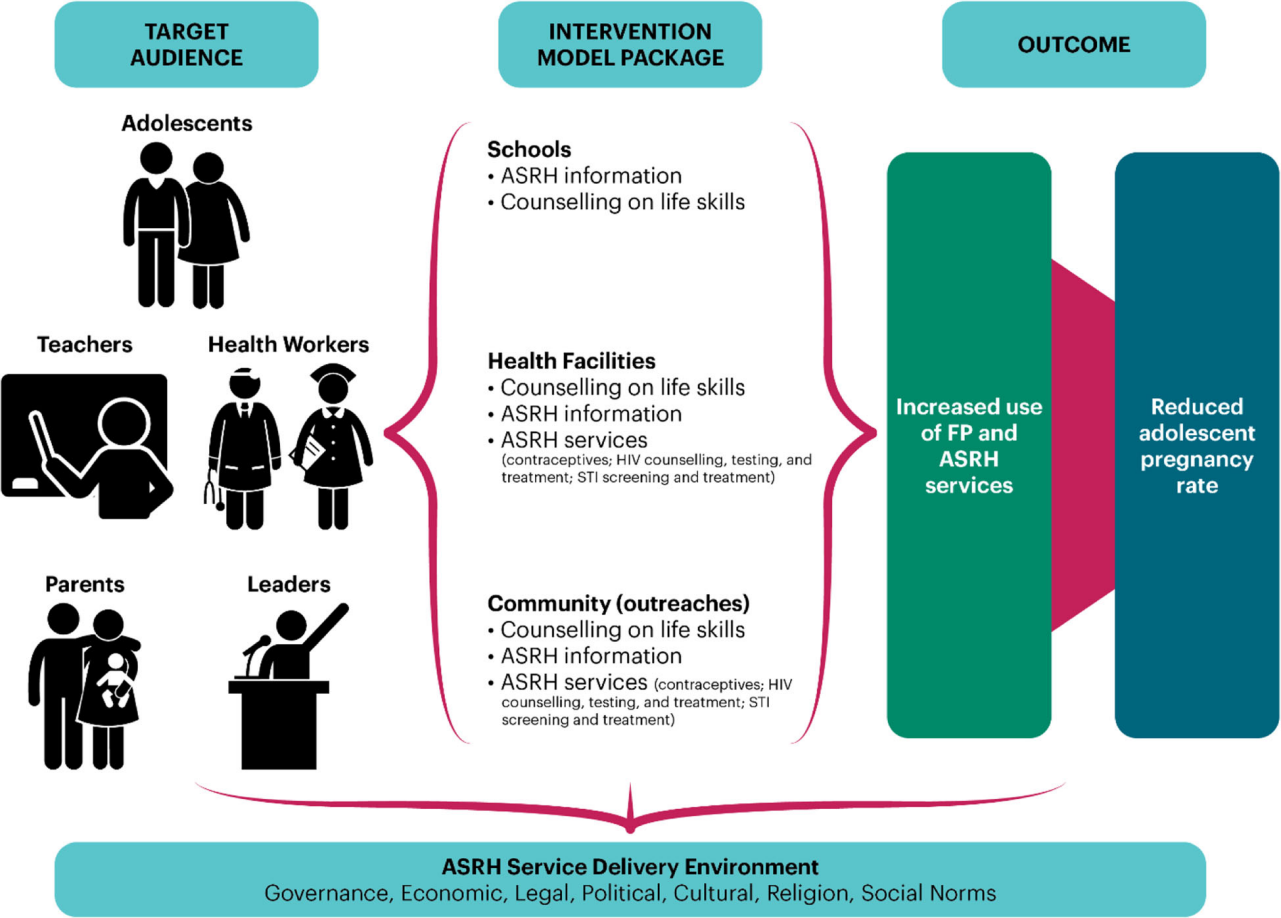
Conceptual framework

The ASRH service package, which will target both boys and girls, will include provision of ASRH services, including counselling and age-appropriate, comprehensive sexual education for behavior change using a multi-sectoral approach with an aim to increase utilization of ASRH services. The intervention will be anchored on the findings of the formative assessment that will seek to explore the ASRH context in the intervention wards. In the comparison group, adolescents will continue to receive the routine ASRH activities provided at facility level. Health facility data quality improvement processes will be implemented in both groups to ensure that ASRH activities are accurately documented. Standard ASRH interventions to be provided are those approved by Ministry of Health and include FP counselling, information and services, HIV counselling, testing and treatment, and screening and treatment for sexually transmitted infections (STIs). The conceptual framework for these interventions is outlined in Fig. 1.

The quality of ASRH services at health facilities at endline will also be assessed through client exit interviews. ASRH service implementation will be through three platforms.

**The community-based component** will involve community health workers and other relevant Community Own Resource Persons (CORPs), such as adolescent counsellors and peer educators, who will be oriented on adolescent-friendly services and other innovative facilitation techniques, such as Education through Listening and Counselling for Choice. The community health workers and CORPs will be supported to carry out household visits and small group sessions and to organize events targeting this cohort such as adolescent camps, symposia, dialogue days, thematic talks, sports days, and monthly facility open days where SRH topics shall be discussed. The sessions will use a targeted approach where services are designed and planned for adolescents alone, are offered in settings that meet the needs of the adolescents, and do not include other groups. During the monthly sessions, health workers will provide ASRH services to willing adolescents after comprehensive counselling. Community resources that are available for adolescents and that can positively contribute to their reproductive health—e.g., drop-in centers, sports facilities, social halls, and bursary funds—will be mapped by the study team led by the Ministry of Interior in collaboration with the Ministry of Health officials.

**The facility-based component** will involve orientation of health workers to provide adolescent-sensitive services. Health workers will be oriented on the values clarification and attitude transformation approach to remove barriers to ASRH services access stemming from misinformation, stigmatization, and negative attitudes toward adolescents seeking services.

**The school-based component** will entail engagement and advocacy sessions targeting the Ministry of Education and county leadership to create buy-in on ASRH rollout in schools. This is critical for schools to allow health workers to train teachers and students on ASRH, offer information, and refer students for services. Thereafter, the intervention schools will be mapped out and teachers trained on selected ASRH modules as recommended by national guidelines. The teachers will then carry out regular (weekly) ASRH sessions in schools and engage students in interactive challenges that build on the new knowledge and skills acquired through the ASRH sessions.

A social behavior change communication approach will be integrated across all service delivery points—i.e., in schools, facilities, and communities. ASRH interventions in schools will include sessions to engage students in interactive challenges that build on their new knowledge and skills acquired through the ASRH sessions. This can be through drama, club meetings, drama debate, music, and sports that will be organized using ASRH themes. Social behavior change communication at health facilities will include whole-site orientation targeting all workers within the participating facilities with information of the package of intervention. Services providers at these facilities will be oriented on recommended ASRH topics, transformation of attitudes, and the provision of adolescent-friendly services. Service providers will also be supported to carry out other interpersonal communication activities, such as facility open days for adolescents and formation of social support structures/groups for this cohort. Participating facilities will be assisted to draft a charter in support of adolescent-friendly services that will provide for, among other things, flexible hours to accommodate the timid, health-seeking behavior of adolescents.

**The virtual platform** is one of the approaches for delivery of ASRH services, but it is not included in this study as the study targets adolescents aged 15–19 years who are primarily in school, living in rural areas, and may not have access to technology platforms. The platform is likely to have more impact among the older youth (19–24 years).

In the comparison group, implementation of ASRH services will continue in the same manner as they are routinely or currently being implemented through health facilities. Health workers will be trained to provide adolescent-friendly services. An endline assessment will be done at the end of the 18 months’ implementation period. The table below presents a summary of the package of interventions that will be done in the two study arms.

The Table 2 presents a summary of the package of interventions that will be carried out in the two study arms.

**Table 2:** Summary of package of activities by evaluation group

	Program intervention group	Comparison group
ASRH service delivery at facility level	✓	✓
ASRH service delivery at community level	✓	✗
ASRH service delivery in schools	✓	✗
Targeted social behavior change communication	✓	✗

### Study outcomes

Our primary outcome will be FP utilization among adolescents as defined as follows:

• *Number of sexually active girls reporting use of modern contraceptive methods*

• *Number of sexually active adolescents aged 15–19 years sampled*

Sexually active girls will be defined as those who report ever having sexual intercourse. Other secondary outcomes will be knowledge and attitude on the use of modern contraception, among others as outlined in Table 3.

**Table 3:** Study outcomes

Other study outcomes among 15–19 years		
Knowledge	Attitude	Practice/utilization
Risks faced by adolescents in pregnancy/childbirth	Toward contraceptives	Utilization of FP services
Available methods of FP	Toward ASRH services offered in facilities/communities/schools	Sexual experience – delay in age at first sex
Secondary study outcomes among 15–19 years: Reduced teenage pregnancy rates in intervention group		

### Data analysis

Data from in-depth interviews, FGDs, and key informant interviews will be transcribed, translated, and back translated then entered in qualitative data software (Atlas ti.8). We will then develop a coding frame using a grounded theoretical framework. Independent analysis will be conducted by different analysts and subsequent comparison between the developed coding matrices will be used to develop a reliability factor for the analysis. The trends from the emerging themes will be iteratively developed by repeatedly analyzing data collected at different time points. Verbatim generated alongside the code matrix will be used to support the emerging thematic framework.

Descriptive analysis of quantitative variables will be done using measures of central tendency (mean, median) and measures of dispersion (range, standard deviation), as appropriate. The inter cluster correlation will be computed. We will then conduct an exploratory analysis to compare outcomes by the sociodemographic, cultural, and economic characteristics (age, education, religion, marital status, life skills, wealth quintile, place of residence [urban or rural]) using the Rao-Scott chi-square test to see if there is any association. To determine the relative importance of these factors on the outcomes, generalized estimating equations, univariate, and multivariate logistic regressions will be used to adjust for clustering within the clusters. Results from the regression analysis will be reported as odds ratio and 95% confidence intervals.

For the quasi-experimental design, our hypothesis is that adolescent girls in the intervention study arm will have greater increases in FP utilization and improved knowledge and attitudes than girls in the control arm. We will estimate the intervention effect by subtracting the baseline FP utilization rate from the estimates at endline then calculating the difference between the comparison and intervention sub-counties (the difference-in-difference analysis). The difference-in-differences analysis assumes a common trend in the outcome in both the intervention and comparison area. We will measure potential confounders at baseline and endline and adjust our analysis for any compositional changes over time in these confounders. All statistical analysis will be considered statistically significant for *p*-values less than 0.05.

### Ethical considerations

In accordance with the principles governing research involving human participants, this study will ensure that respondents’ ethical rights are upheld. Ethical approval has been granted by the Kenya Medical Research Institute (number 651) and the Johns Hopkins University School of Public Health number (9227) institutional review boards. Approval by the county health management team and the health facility in-charges has also been granted. All adult participants will be required to give an informed written consent prior to participating in the study. Consent will be indicated by a signature or thumb print on the form. Parental permission for adolescents aged 15–17 years will be sought first, before the minor’s assent is sought. No minor will participate in the study until permission is provided by a parent/guardian and informed assent obtained from the minor. In addition, the data collectors and qualitative researchers will be experienced research staff who have a minimum of post-secondary education (college diploma) and who will undergo training on basic research ethics and study procedures, including maintaining confidentiality.

### Study status

Table 4 outlines the timeline for the study.

**Table 4:**
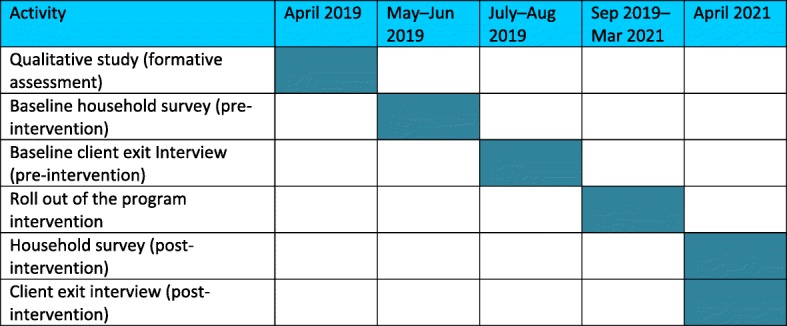
Study timeline

### Dissemination of study findings

Dissemination will target multi-sectoral stakeholders and will be done at sub-county, county, and national level. This will include ministries of health, education, gender, and children’s services. The Ministry of Interior and Coordination of National Government, which is responsible for the police and administration, will also be involved. The results will be published in peer-reviewed journals and presented at national and international conferences.

## Discussion

Adolescent sexual reproductive health has gained traction in recent years. Sustainable Development Goal 3 aims to ensure universal access to SRH services by 2030, including FP, information and education, and the integration of reproductive health into national strategies and programs [11]. In addition, adolescents have now been included in the World Health Organization’s *Global strategy for women’s, children’s and adolescents’ health* (2016–2030), acknowledging the unique health challenges facing young people and their pivotal role, alongside women and children, as key drivers of change in the post-2015 era [12]. In Kenya, there have been milestones in policy regarding ASRH, however, a 2013 assessment of the 2003 policy revealed that there was limited dissemination of the policy and that it was not implemented equitably to all adolescents, especially the hardest to reach and most vulnerable populations [13]. In addition, barriers continued to exist that affect access to adolescent-friendly SRH services. As such, access to and utilization of ASRH services remains a challenge.

This study will evaluate the effectiveness of a combined approach to increase utilization of ASRH services. Results of the qualitative analysis and discussions with various stakeholder—such as teachers, health workers, and community representatives—will provide information on barriers to and enablers of use of ASRH services and the study team will develop recommendations on how to address the barriers and support enabling factors. The study will also provide information on the quality of ASRH services offered at health facilities, schools, and communities (outreaches) and provide recommendations on how services can be improved. Implementation of the study and results obtained from this study will be of direct benefit to the study sites and will contribute to improved utilization of ASRH services and reduction of teenage pregnancy in Kisumu and Kakamega counties. In additional, the study will generate evidence on whether a combined approach that targets several sectors—health, education, youth, and administration—works to improve utilization of ASRH services. The information generated from the study will also be beneficial for programming as it will identify underlying reasons as to why access to and utilization of ASRH services is low. Results will be helpful in improving programs that aim to address ASRH and reduce teenage pregnancy within the Kenyan context and in other similar low middle-income countries.

## Data Availability

Not applicable.

## Abbreviations

ASRH: Adolescent sexual and reproductive health
CORPS: Community Own Resource Persons
FGD: Focus group discussion
FP: Family Planning
IDI: In-depth interview
KII: Key informant interview
MOH: Ministry of Health
SRH: Sexual and reproductive health
STI: Sexually transmitted infections
